# Draft genome sequences of 13 *Salmonella enterica* subsp. *enterica* isolates from chickens, cows, and Canadian *Salmonella* outbreaks

**DOI:** 10.1128/mra.00989-23

**Published:** 2024-04-30

**Authors:** Jinha Suh, Bridget O'Brien, Paul D. Glenn II, Zhangbin Cai, France Daigle, Sébastien P. Faucher, Jennifer Ronholm

**Affiliations:** 1 Faculty of Agricultural and Environmental Sciences, Macdonald Campus, McGill University, Sainte-Anne-de-Bellevue, Québec, Canada; 2 Département de Microbiologie, Infectiologie et Immunologie, Université de Montréal, Montréal, Québec, Canada; 3 Centre de Recherche en Infectiologie Porcine et Avicole (CRIPA), Faculté de médecine vétérinaire, Saint-Hyacinthe, Québec, Canada; University of Maryland School of Medicine, Baltimore, Maryland, USA

**Keywords:** *Salmonella*, OneHealth

## Abstract

*Salmonella enterica* is the etiological agent responsible for salmonellosis. Here, we report the draft whole genome sequences of 13 *S*. *enterica* subsp. *enterica* isolates from chickens and cows, as well as from previous Canadian *Salmonella* outbreaks investigated by the Canadian Food Inspection Agency.

## ANNOUNCEMENT

*Salmonella enterica* is a zoonotic pathogen that is responsible for serious infections in animals and humans. More than 2,500 serovars have been identified from a wide range of hosts, including chickens, cows, and humans (1–3). *S. enterica* ser. Gallinarum biotypes Pullorum and Gallinarum target avian hosts, resulting in severe, life-threatening diseases (1, 3). *S. enterica* ser. Dublin infects cattle and can lead to reduced milk production and spontaneous abortions, while in humans it can lead to a systemic infection often requiring hospitalization (4). *S. enterica* ser. Enteritidis is a broad host range pathogen that can infect chicks, calves, and humans (4). This project was undertaken to sequence a variety of *S. enterica* serovars primarily associated with agricultural production in Canada. The whole genome sequences produced will be used to guide the construction of deletion knockout mutants that will be used in future studies to help understand host-species specificity in various *S. enterica* serovars.

The Animal Health Microbiology Laboratory within the Canadian Food Inspection Agency (CFIA)/Ottawa Laboratory (Fallowfield) maintains a historical culture collection of *Salmonella* isolates that were collected from previous *Salmonella* outbreaks in Canada (5). *S. enterica* ser. Gallinarum biotype Pullorum, *S. enterica* ser. Gallinarum biotype Gallinarum, and *S. enterica* ser. Enteritidis strains sequenced here were generously donated from the CFIA collection. *S. enterica* ser. Dublin strains were generously donated by the Centre de diagnostic vétérinaire de l’Université de Montréal.

*S. enterica* isolates were stored at −80°C and reanimated by overnight culture on tryptic soy agar (TSA) at 37°C. A single colony was selected from the TSA plate and grown in tryptic soy broth at 37°C with agitation at 200 rpm overnight. The DNA UltraClean Microbial Kit (Qiagen, Hilden, Germany) was used along with 1.8 mL of liquid culture to extract bacterial DNA. Sequencing libraries were prepared using the Nextera Flex DNA Library Preparation Kit (Illumina, San Diego, CA, USA) and Nextera DNA CD indexes (96 indexes, 96 samples) according to the manufacturer’s instructions. Samples were pooled to equimolar concentrations and sequenced using paired-end libraries on a MiSeq benchtop sequencer (Illumina) for 301 cycles in each direction, generating 2,786,915 reads. Reads were assembled *de novo* into high-quality draft genomes with ProkaryoteAssembly (v.0.1.6) (https://github.com/BFSSI-Bioinformatics-Lab/ProkaryoteAssembly). Default parameters were used except for the trimming step, for which the command trimq=20 2> {stats_out} was applied on low-quality sequences (*Q* score <20). Nonoverlapping contiguous sequences were generated, and contigs <1,000 bp were removed. The species and serovar of each isolate were confirmed using GTDBTk (v.2.3.2) (6) and SeqSero2 (v.1.2.1) (7). Gene predictions and annotations were performed using Prokka (v.1.14.5) (8). The contiguity of assemblies and N50 values were measured using QUAST (v.4.4) (9). Default parameters were used except where otherwise noted.

**TABLE 1:** Sequencing and annotation information for *S. enterica* isolates

Strain identification no.	Serovar	SRA accession no.	GenBank accession no.	Draft genome size (bp)	Coverage (×)	GC content (%)	No. of contigs	No. of CDSs^*a*^	No. of RNAs^*b*^	N50
D3-SHY-2021-534	Dublin	SRR25996677	JAVQGX000000000	4,948,677	119	51.86	25	4,763	77	420,976
D4-SHY-2021-988	Dublin	SRR25996676	JAVQGY000000000	4,898,470	59	51.85	29	4,710	77	478,460
D5-SHY-2021-1051	Dublin	SRR25996675	JAVQGZ000000000	4,967,813	64	51.88	27	4,787	77	420,978
F7-SHY-2021-4385	Dublin	SRR25996674	JAVQHA000000000	4,916,336	47	51.95	25	4,725	74	401,747
G1-SHY-2021-5399	Dublin	SRR25996673	JAVQHB000000000	4,966,336	50	51.83	30	4,780	76	327,339
01D587s.K-6	Gallinarum biotype Pullorum	SRR25996672	JAVQHC000000000	4,738,594	81	51.92	25	4,575	67	405,529
903541	Gallinarum biotype Pullorum	SRR25996671	JAVQHD000000000	4,741,239	76	51.92	29	4,575	68	510,299
Poppe #514	Gallinarum biotype Pullorum	SRR25996670	JAVQHE000000000	4,637,895	43	51.94	22	4,446	66	510,173
2B-015.5	Gallinarum biotype Gallinarum	SRR25996683	JAVQHF000000000	4,698,974	49	51.85	22	4,549	66	510,429
Poppe #508	Gallinarum biotype Gallinarum	SRR25996682	JAVQHG000000000	4,674,161	70	51.88	25	4,526	67	400,620
PT 8	Enteritidis	SRR25996680	JAVQHI000000000	4,690,948	58	51.86	19	4,395	76	489,740
PT 9b	Enteritidis	SRR25996679	JAVQHJ000000000	4,721,133	48	51.81	26	4,455	73	433,290
PT 13	Enteritidis	SRR25996678	JAVQHK000000000	4,723,670	75	51.91	20	4,438	74	489,889

^*a*^ CDSs, coding sequences.

^*b*^ RNAs, sum of tmRNA and tRNA counts.

## Data Availability

These nucleotide sequences have been deposited in DDBJ/ENA/GenBank as BioProject PRJNA1014929 under the accession numbers provided in Table 1. The annotated genome assemblies are made public through FigShare (https://doi.org/10.6084/m9.figshare.25137752.v1).
